# Release of a Wound-Healing Agent from PLGA Microspheres in a Thermosensitive Gel

**DOI:** 10.1155/2013/387863

**Published:** 2013-10-03

**Authors:** H. A. Machado, J. J. Abercrombie, T. You, P. P. DeLuca, K. P. Leung

**Affiliations:** ^1^US Army Dental and Trauma Research Detachment, Institute of Surgical Research, 3650 Chambers Pass, Building 3610, JBSA Fort Sam Houston, TX 78234-6315, USA; ^2^Faculty of Pharmaceutical Sciences, University of Kentucky College of Pharmacy, Lexington, KY 40506, USA

## Abstract

The purpose of this research was to develop a topical microsphere delivery system in a thermosensitive 20% poloxamer 407 gel (Pluronic F127) to control release of KSL-W, a cationic antimicrobial decapeptide, for a period of 4–7 days for potential application in combat related injuries. KSL-W loaded microsphere formulations were prepared by a solvent extraction-evaporation method (water-oil-water), with poly (D,L-lactic-co-glycolic acid) (PLGA) (50 : 50, low-weight, and hydrophilic end) as the polymeric system. After optimization of the process, three formulations (A, B, and C) were prepared with different organic to water ratio of the primary emulsion while maintaining other components and manufacturing parameters constant. Formulations were characterized for surface morphology, porous nature, drug loading, *in vitro* drug release, and antimicrobial activity. Microspheres containing 20% peptide with porous surfaces and internal structure were prepared in satisfactory yields and in sizes varying from 25 to 50 **μ**m. Gels of 20% Pluronic F127, which were liquid at or below 24.6°C and formed transparent films at body temperature, were used as carriers for the microspheres. Rheological studies showed a gelation temperature of 24.6°C for the 20% Pluronic F127 gel alone. Gelation temperature and viscosity of formulations A, B, and C as a function of temperature were very close to those of the carrier. A Franz diffusion cell system was used to study the release of peptide from the microspheres suspended in both, phosphate-buffered saline (PBS) and a 20% Pluronic F127 gel. *In vitro* release of greater than 50% peptide was found in all formulations in both PBS and the gel, and in one formulation there was a release of 75% in both PBS and the gel. Fractions collected from the release process were also tested for bactericidal activity against *Staphylococcus epidermidis* using the broth microdilution method and found to provide effective antimicrobial activity to warrant consideration and testing in animal wound models for treating combat-related injuries.

## 1. Introduction

Combat-related injuries during military operations have been associated with infectious complications due to the nature of wounding, giving rise to significant devitalized tissue, contamination of battlefield wounding agents with various bacteria, and the emergence of multidrug-resistant nosocomial pathogens, especially gram-negative bacteria. Investigations are underway to identify the source of these bacteria and to try to mitigate their associated morbidity and mortality. As in the US civilian medical community, new antimicrobial agents are needed to treat these infections. Platforms to identify infection and its antimicrobial resistance profile also are needed to ensure that appropriately focused therapy is instituted [1].

Cationic antimicrobial peptides are excellent candidates for development as novel therapeutic agents to complement conventional antibiotic therapy [2]. In contrast to conventional antibiotics, antimicrobial peptides have lower propensity to induce antibiotic resistance. These peptides generally exhibit a broad range of bactericidal activity which requires a short contact time to induce killing. However, many available peptide and protein drugs are characterized by short biological half-lives. They are easily degraded by enzymes and pass poorly through biological barriers because of deficient diffusivity and low partition coefficient [3–6]. The assessment of protein stability in delivery systems is increasingly being integrated into research programs. Among the various means used, nanospheres or microspheres made of poly (D,L-lactic-co-glycolic acid) (PLGA) have gained popularity, mainly because of their tissue compatibility and biodegradability [7]. Poly (lactic-co-glycolic acid) (PLGA) is made from two monomers, lactic acid and glycolic acid. The ratio of the monomers and the molecular weight set the identity and properties. PLGA is both biodegradable and biocompatible, and since both monomers occur naturally it has minimal toxicity. PLGA is naturally amorphous (not crystalline). While poly (lactic) and poly (glycolic) acid are poorly soluble in most solvents; PLGA dissolves in many common solvents including tetrahydrofuran, acetone, ethyl acetate, and chlorinated solvents. The Food and Drug Administration (FDA) has approved PLGA for preparation of micro- and nanoparticles.

Peptide encapsulation and preparation of PLGA microspheres are generally performed using a water-in-oil-in-water double-emulsion technique. In this process, the active ingredient is first dissolved in an aqueous phase (W), which is then emulsified in an organic solvent (O) of a polymer to make a primary W/O emulsion. This primary emulsion is further mixed in an emulsifier-containing aqueous solution (W) to make a W/O/W double emulsion. The removal of the polymer solvent leaves microspheres in the aqueous continuous phase, making it possible to collect them by filtering or centrifuging [8–10]. Incorporating the drug-loaded microspheres into a thermoreversible gel would provide a novel platform in the treatment of persistent infections arising from localized biofilms [11–13].

Microspheres with high peptide incorporation efficiency can be prepared from low molecular weight and hydrophilic acid ended PLGA polymers [14]. As a vehicle or carrier, Pluronic F-127, in concentrated solutions (>18% in water), can be transformed from low-viscosity transparent solutions to solid gels on warming to body temperature [15, 16]. This phenomenon, therefore, suggests that when poured or sprayed onto the skin, the gel preparation will form a semisolid artificial barrier and a sustained release depot.

Pluronic F-127 consist of ethylene oxide (EO) and propylene oxide (PO) blocks arranged in a triblock structure (EO)_*x*_-(PO)_*y*_-(EO)_*x*_; the chemical formula is HO[CH-CH_2_O]_*x*_[CH(CH_3_CH_2_O)]_*y*_ where *x* = 95–105 and *y* = 54–60. It has a molecular weight of about 12,600 (9,840–14,600). The gelation mechanism of pluronic solutions indicates a micellar mode of association. Micelle formation occurs at the critical micellization temperature as a result of polypropylene oxide block dehydration. With the increasing of temperature, micellization becomes more important and, at a certain temperature, micelles come into contact and no longer move. This packing of micelles and micelle entanglement might be the possible mechanism of pluronic solution gelation with increase of temperature.

The purpose of the present work was to develop a thermosensitive gel loaded with biodegradable microspheres that release an antimicrobial peptide up to one week. The microspheres were manufactured using a water-in-oil-in-water double-emulsion solvent evaporation technique. By monitoring the organic to water ratio of the primary emulsion while maintaining constant other manufacturing parameters, desired porosity of microspheres that fill the pharmacokinetic requirements may be achieved [17].

## 2. Materials and Methods

KSL-W (sequence KKVVFWVKFK-CONH2), lot no. 05US1311-B, MW 1307, was custom synthesized by Synprep (Dublin, CA); PLGA 502H (poly (D,L-Lactide-co-glycolide 50 : 50 MW 11,300, lot no. STBB9327V); pharmaceutical grade polyvinyl alcohol low-viscosity, lot no. MKBD9933V, MW: 31,000–50,000, 98%-99% hydrolyzed were obtained from Sigma; Lutrol F127 (Pluronic F127), lot no. WPTF531B, was provided by Q.BASF; Mueller Hinton broth (Becton, Dickinson and Company, Spark, MD). All other chemicals used were analytical grade and were obtained from Sigma.

### 2.1. KSL-W Assay

**Figure 1:**
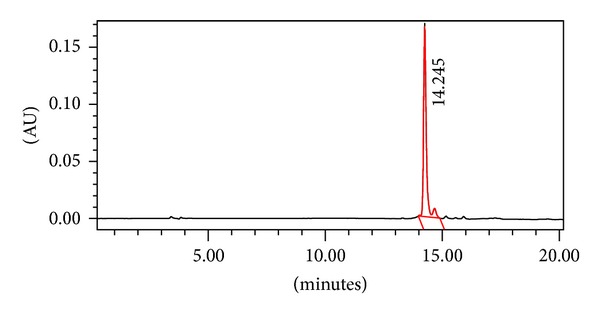
High-performance liquid chromatogram of KSL-W (0.5 mg/mL) in DI water.

**Figure 2:**
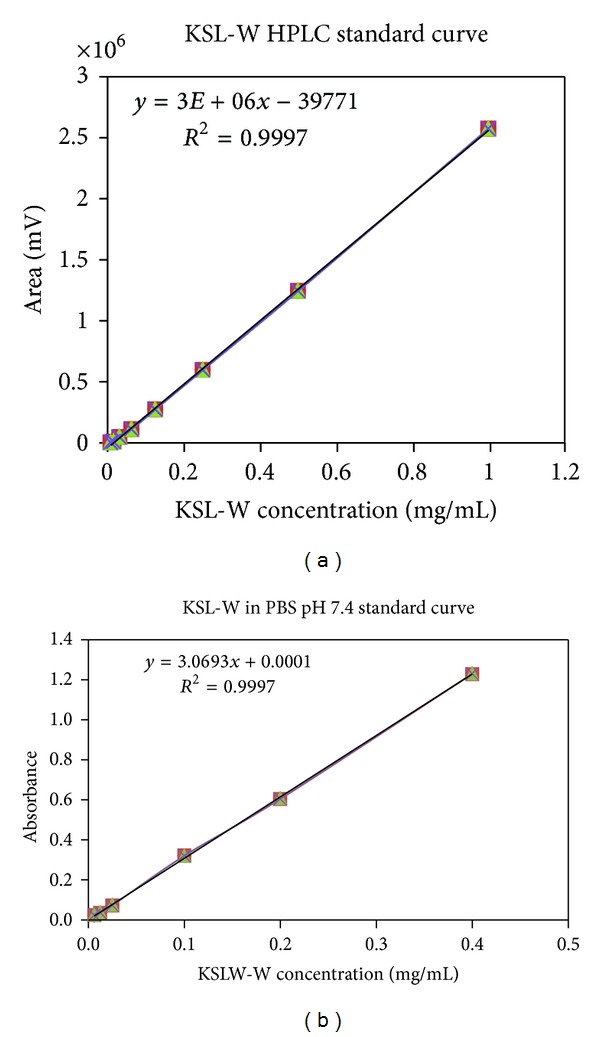
KSL-W standard curves (280 nm). (a) high-performance liquid chromatography; (b) UV spectrophotometry. Readings were obtained from samples determined in triplicates (mean ± standard errors).

KSL-W content of microspheres was determined by reverse-phase high-performance liquid chromatography (HPLC) using a Waters 600 E multisolvent delivery system consisting of a Waters 600 pump and controller, a Waters 717 plus autosampler, and a Waters 996 photodiode array detector. The column used was a Prosphere C18-300 (4.6 × 250 mm, 5 *μ*m). A gradient elusion was accomplished with acetonitrile : water : trifluoroacetic acid 10% : 90% : 0.1% (A) and acetonitrile : water : trifluoroacetic acid 70% : 30% : 0.1% (B) and increasing the amount of B from 0 to 100% over 20 minutes at a flow rate of 1.0 mL/min. Each sample was run for 35 min with a 5 min lag time between samples. KSL-W shows a peak at a retention time of 14.245 min (Figure 1). Standard curves of KSL-W ranging from 6.25 *μ*g/mL to 1 mg/mL yielded linear responses over the concentration range with detection at 280 nm (Figure 2(a)). KSL-W release from microspheres incorporated in the gel was determined by UV spectrophotometry at 280 nm using an Eppendorf Biophotometer (Figure 2(b)).

### 2.2. Preparation of Microspheres

PLGA microspheres loaded with KSL-W were prepared by using the double-emulsification solvent evaporation method (W/O/W) [18–20], with PLGA 50 : 50, low-weight, and hydrophilic end, as the polymeric system. Three formulations having different organic to water ratio of the primary emulsion were prepared. Other conditions and variables applied for the preparation of KSL-W microspheres were maintained constant. Formulations composition is shown in Table 1.

**Figure 3:**
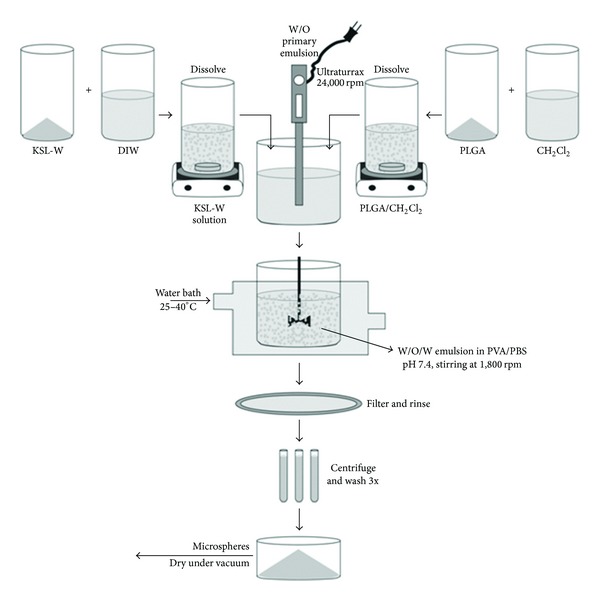
Schematic of the double-emulsification solvent extraction/evaporation method for the preparation of KSL-W-PLGA microspheres. Polycarbonate (PC) membrane filter (1 *μ*m size cutoff) was used to filter the MS.

KSL-W was dissolved in deionized water and Tween 20 was added. The PLGA was dissolved in dichloromethane (CH_2_Cl_2_). The KSL-W solution was slowly added to the PLGA solution to form a primary W/O emulsion by stirring with an Ultraturrax homogenizer at 24,000 rpm for 5 minutes. The primary emulsion was slowly poured into 200 mL of 10× phosphate-buffered saline (10×-PBS), pH 7.4 containing 0.20 or 0.35% polyvinyl alcohol (PVA), and stirred at 1,800 rpm (overhead propeller) for sufficient time to form microspheres. This allowed the CH_2_Cl_2_ to evaporate prior to filtration and rinsing.  Figure 3 depicts this evaporation method for preparing microspheres. Following filtration and rinsing, the solidified microspheres were transferred from the filter to centrifuge tubes, washed three times with DIW, centrifuged at 2,500 rpm for 10 min, and dried at 25°C under vacuum overnight.

### 2.3. Characterization of KSL-W/PLGA Microspheres

The characterization focused on comparing the porosity of the microspheres produced, and correlating such porosity to the *in vitro* release profiles of the final formulations. Determining how this porosity affected other MS parameters (surface morphology, particle size, drug content, loading efficiency %, Yield %) allowed a selection of the formulation(s) that guaranteed bactericidal activity of the delivery system for at least one week. The antimicrobial activity of the release fractions collected for each KSL-W microsphere formulation was determined against *Staphylococcus epidermidis*.

#### 2.3.1. Microsphere Surface Morphology and Particle Size

Scanning electron microscopy (SEM) (Zeiss Field Emission, Sigma VP-40, Germany) was used to characterize the surface morphology of microspheres. Microspheres were mounted directly on the SEM stub using double stick carbon tape, coated with gold/palladium, and scanned in a high-vacuum chamber with a focused electron beam. Secondary electrons emitted from the sample were detected and the image was formed. The average particle size and size distribution were determined by using Smart Tiff from Zeiss.

#### 2.3.2. Drug Content (KSL-W Content in the Microsphere Powder) (See [21])

Fifty mg of peptide loaded microspheres were quantitatively weighed and dissolved in 2 mL of CH_2_CL_2_, and the peptide was extracted with 10 mL of 0.1 M acetate buffer with a pH of 4.0 by shaking for 1 hour in a wrist-action shaker. The aqueous buffer phase was separated by centrifugation, and the extracted peptide concentration was determined by reversed-phase HPLC

(1) KSL-W  in  MS  powder(%  w/w)   =[Cb(mg/mL)×10 mL50 mg]×100Cb=KSL-W  concentration  in  acetate  buffer  (mg/mL).

#### 2.3.3. Loading Efficiency (%)

(2) Loading  efficiency(%) =  (total  amount  of  KSL-W  in  microspheres)×100Amount  of  KSL-W  initially  weighed.

#### 2.3.4. Yield (%)

Obtained microspheres were weighed, and percentage yield was determined by using the following formula:

(3) Yield  (%) =(weight  of  MS  obtainedtotal  weight  of  KSL-W  and  PLGA  used)×100.

### 2.4. Preparation and Rheological Tests of Thermosensitive Formulations

#### 2.4.1. Preparation of Concentrated Pluronic (F127) Solution (See [22])

Aqueous 22.2% pluronic F127 w/v was prepared by the cold method described by Schmolka [22]. The method involved slow addition of polymer in cold water with continuous agitation. The mixture was stored overnight at 4°C to obtain a clear solution.

#### 2.4.2. Incorporation of MS in Pluronic (F127) Solution

Dried microspheres were resuspended in 2 mL distilled water. This suspension was added to the cold concentrated pluronic (F127) gel so that the final concentration of pluronic reached 20% w/v and gently stirred for 10 min for uniform distribution of MS in the pluronic.

#### 2.4.3. Gelation Temperature (Tg) Measurement (See [23])

Ten mL of cold formulation and a magnetic stir bar were placed into a 25 mL glass vial that had been placed in a low-temperature water bath. A thermometer probe was immersed into the sample solution. The temperature was slowly increased (2°C/min) under continuous stirring. Temperature at which the magnetic bar stopped moving was reported as the gelation temperature (Tg).

#### 2.4.4. Viscosity Measurement (Centipoises, cP)

Formulation viscosities were measured using a low viscosity Brookfield viscometer (model LVDV-E-115, Cole Palmer, Vernon Hills, IL, USA) with a small sample volume adapter and spindles no. 61 (for low viscosity) and no. 34 (for higher viscosity). Viscosity of formulations was measured at seven different temperatures. These included the gelation temperature (Tg), four temperatures below the Tg, and two temperatures above the Tg. The principle of operation of the DV-E viscometers is to rotate a spindle (immersed in the test fluid) through a calibrated spring. The viscous drag of the fluid against the spindle is measured by the spring deflection in centipoises (cP) or millipascal seconds (mPa·s). 1 cP = 1 mPa·s.

### 2.5. *In Vitro* Release Profile and Bactericidal Activity of Final Formulations

#### 2.5.1. *In Vitro* Release Profile (See [24–26])

*In vitro* release profile of KSL-W from the PLGA microspheres suspended in PBS and in 20% Pluronic F127 gel was performed in triplicate for each formulation using a six-station Franz diffusion cell. A half of a mL of each microsphere suspension was placed into each corresponding donor chamber of the instrument. The donor chamber was separated from the acceptor chamber by a 0.1-*μ*m polycarbonate (PC) membrane. The medium solution in the acceptor chamber was 5 mL PBS at pH 7.4. Acceptor chamber was kept at 37°C (circulating water) and was continuously stirred (magnetic stirrer). Release samples of 0.2 mL were removed at different intervals (1, 2, 4, 6, and 12 hours and 1 to 7 days) from the acceptor chamber. The volume of the sample removed was replaced with the same volume of fresh buffer after each sampling. KSL-W content was evaluated by a UV-Vis spectrophotometer at 280 nm, and the amount of KSL-W was determined by extrapolation in a previously prepared calibration curve.

#### 2.5.2. Bactericidal Activity (See [27])

Bactericidal activity was determined against *Staphylococcus epidermidis* using the broth microdilution method. Freshly grown cultures of *Staphylococcus epidermidis* at exponential phase were used as the inoculum. Bacteria were centrifuged at 4,000 rpm for 15 min at 4°C, suspended in 2× Mueller Hinton broth, and adjusted to approximately 4 × 10^6^ colony-forming units (cfu) per milliliter in 2× Mueller Hinton broth. Release sample (100 *μ*L) was added to each well of a 96-well, flat-bottomed plate (Becton Dickinson, Spark, MD, USA). The solution was serially diluted (twofold) with sterile distilled water in the wells. Aqueous KSL-W peptide at 200 *μ*g per mL, which served as the positive control, was included in the assay and serially diluted in sterile distilled water as the release sample. After dispensing 100-*μ*L aliquots of bacterial suspension into the wells, the 96-well plates were incubated at 37°C for 24 hours. The minimum inhibitory concentration (MIC) was defined as the lowest concentration of the release sample (containing released peptide) or KSL-W peptide solution that prevented visible turbidity, as measured at 600 nm by using an ELISA reader (BIOTEK, Winooski, VT, USA).

## 3. Results and Discussion

Table 1 shows that differences in composition of formulations A, B, and C are only in the amount of water used to dissolve the peptide in the O/W primary emulsion. Other variables and manufacturing parameters have remained constant; therefore, we can assume that differences in properties of the obtained MS are due to differences in the organic to water (O/W) ratio of their initial composition.

**Figure 4:**
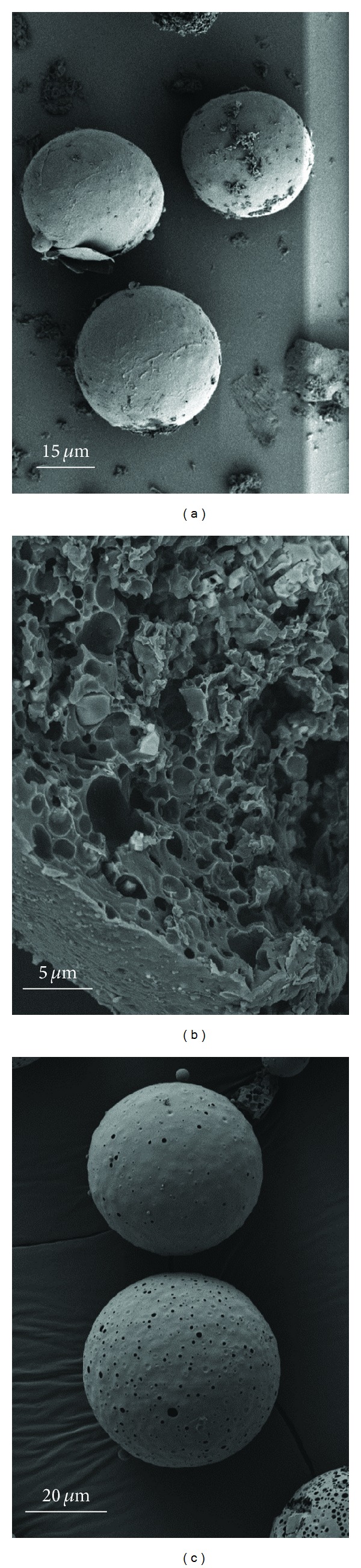
SEM images of microsphere Formulation A.

**Figure 5:**
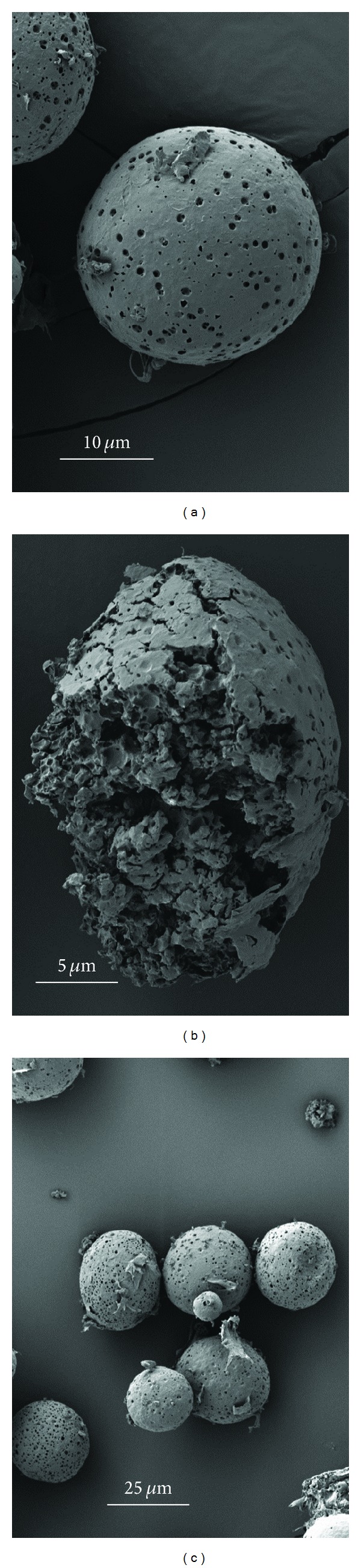
SEM images of microsphere Formulation B.

**Figure 6:**
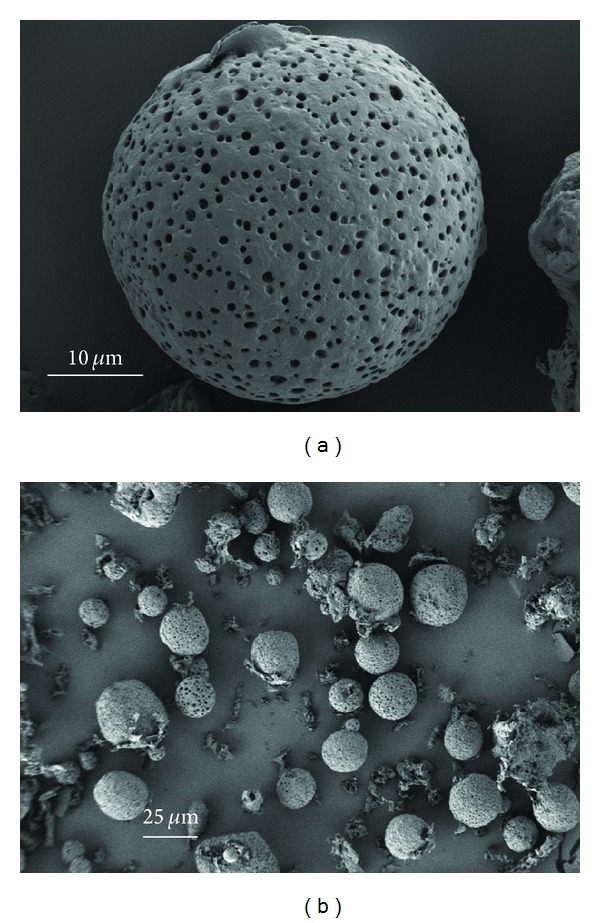
SEM images of microsphere Formulation C.

The scanning electron micrographs (SEMs) are shown in Figures 4, 5, and 6. The microparticles were spherical for the three formulations and essentially free of aggregation. Surface morphology showed smooth surfaces with varying porosity from low for Formulation A to high for B and C, with C being the highest. Fractured particles of A and B reveal a very porous interior.

**Figure 7:**
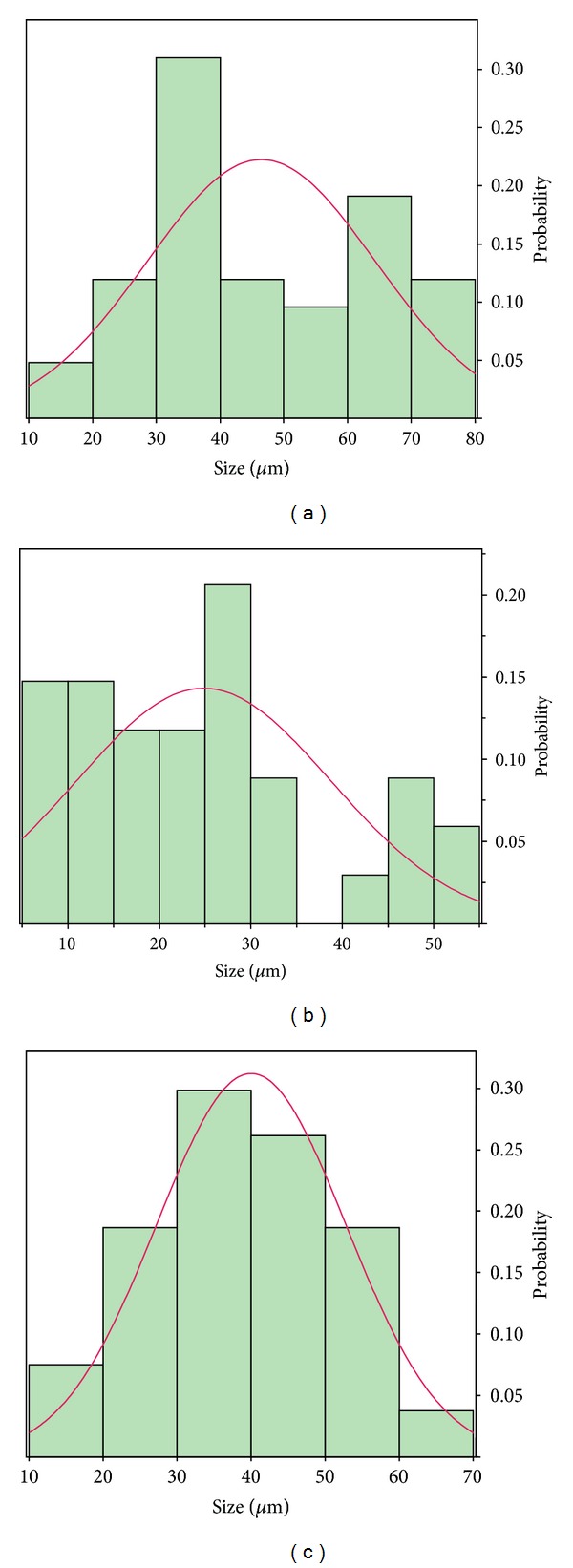
KSL-W PLGA microspheres particle size and particle size distribution using Smart Tiff from Zeiss;  *n* = 30. Average means; Formulation A, 46.4 *μ*m ± 17.9 *μ*m; Formulation B, 25.8 *μ*m ± 14.1 *μ*m; Formulation C, 40.0 *μ*m ± 12.8 *μ*m.

The properties and characteristics of the peptide-loaded PLGA microspheres are tabulated in Table 2. Yields ranged from 68 to 84% with loading efficiencies of 68–78% and drug contents of 17–20%. The average particle size as determined from the SEMs was 46.42 *μ*m ± 17.9 *μ*m for Formulation A (Figure 7(a)), 25.8 *μ*m ± 14.1 *μ*m for 02–15 (Figure 7(b)) and 40 *μ*m ± 12.8 *μ*m for C (Figure 7(c)). Surface area measurements were not performed but formulation A, due to larger particle size, would have the lowest surface area, while C would have a higher surface area than A but lower than B which has the smallest particle size.

**Figure 8:**
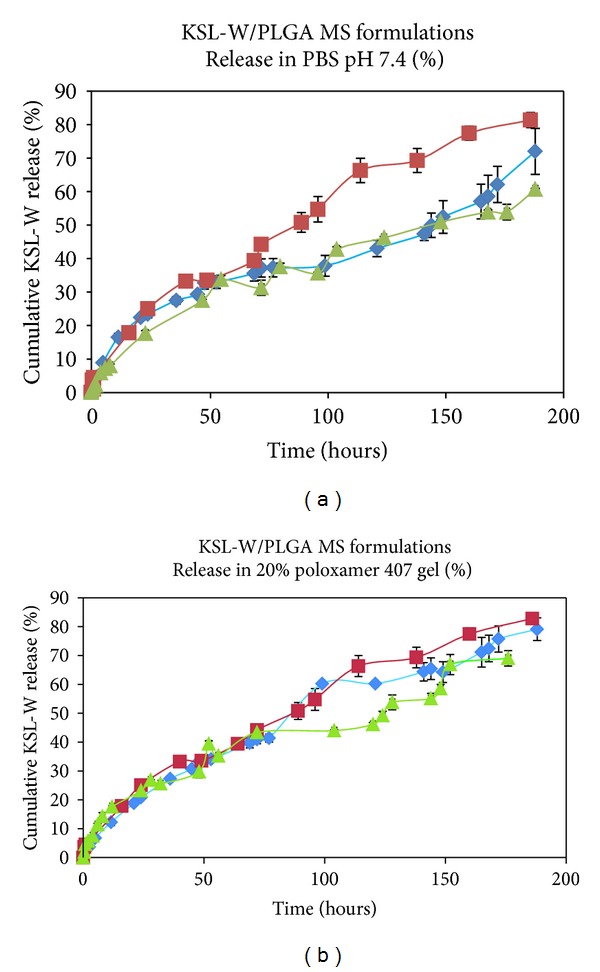
*In vitro *peptide release test utilizing the Franz diffusion cell. Formulation A (▲); Formulation B (■); and Formulation C (♦). In all samples,  *n* = 3. Membrane filter: 0.1 *μ*m PC. Acceptor media: 5 mL PBS pH 7.4. Sampling volume: 0.2 mL. Sample size in donor chamber: 0.5 mL.

*In vitro* release of KSL-W from the microspheres suspended in PBS and in 20% Pluronic 127 is listed in Table 2 at 150 hours for comparative purposes, since a delivery system lasting 4 to 7 days in a wound is necessary. As shown in Figure 8, more than 60% was released in 150 hours for the three formulations with B showing the highest release, 74.8% in PBS and 76.3% in the gel. There is no detectable burst release difference of MS formulations in Pluronic F127 20% gel. But the release for A decreased considerably after 96 hours in this medium. The subsequent slower rate is understandable considering the larger particle size and lower porosity for A, giving rise to lower specific surface area and a resulting lower polymer degradation rate.

Release of the peptide from the microsphere is controlled by two mechanisms: diffusion of the protein out of the microsphere and erosion of the polymer matrix. Typically, the diffusion process consists of an initial “burst” release of peptide at or near the surface of the microsphere followed by the additional release of protein from the pores of the microsphere. Erosion occurs by hydrolysis of the polymer matrix generating pores that expose interior pockets of peptide to the bathing liquid. For continuous release, the diffusion and erosion process must balance each other to allow the peptide to diffuse out of the microsphere at a constant rate.

The formulations produced in this study did not yield a complete release of the peptide. A number of reasons could cause the incomplete release when double-emulsion/solvent evaporation method is being used as in this study. For example, one of the possible causes for the incomplete release observed might be due to nonspecific adsorption of peptides to interfaces or to microsphere materials. In addition, the peptide could potentially be denatured or degraded when exposed to a variety of stress conditions including heat, shear, and organic solvents exposure during the preparation of microspheres.

Even though not very high in our case, in general, hydrophilic polymers in the formulations result in a high initial burst and high release rates when PLGA with relatively high glycolide content (50/50) is used [28]. The higher glycolide content makes the polymer more hydrophilic and facilitates water uptake from the release medium. The PLGA used in the formulations (D,L-Lactide-glycolide 50 : 50, acid terminated, MW 7,000 to 17000 from Sigma) corresponds to Resomer RG 502 H, a PLGA carrying free carboxylic end groups that resulted in a higher incorporation efficiency, as well as a higher hydration, as compared to the end-capped polymer [29].

Release from both B and C microsphere formulations was continuous with B showing a similar release profile in both PBS and the Pluronic gel. What is difficult to explain is the similar release for B in PBS and in the gel, while for C release was greater in the gel. Evidently, hydration in the gel may be greater or the pluronic may accelerate the polymer degradation. Nevertheless, a release of 75% at 150 hours from the B microspheres in both PBS and the Pluronic gel suggests that this is the preferable formulation to pursue as a wound-healing device.

**Figure 9:**
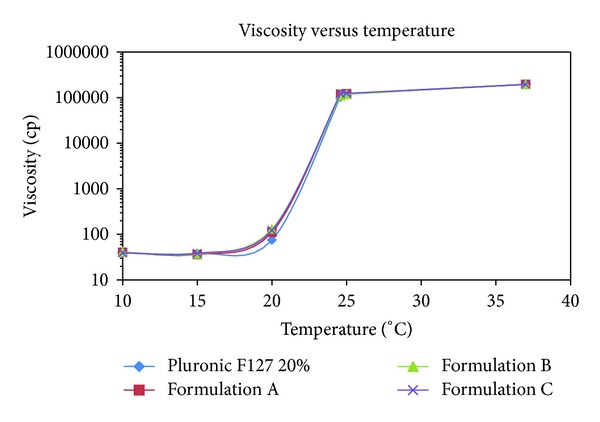
Viscosity profiles of formulations prepared from pluronic F127 as a function of temperature. Formulation A (▲); Formulation B (■); and Formulation C (♦); Pluronic F127 20% Blank (•). All test formulations and the blank carrier display similar viscosity profiles as a function of the temperature. Readings (mean ± standard deviations) are from samples tested in triplicates.

Table 3 and Figure 9 show that the gelation temperature and viscosity of formulations A, B, and C were very close to those of the blank formulation (pluronic F127 20% solution containing no MS powder) suggesting that MS powder did not interfere with the rheological properties of the carrier. Ideally an *in situ* gelling system used in an open wound or burned skin should be low in viscosity as a free flowing liquid to allow for easy and accurate topical administration to the injured site. The gel formed following phase transition at body temperature should be strong enough to remain on the site and exhibits a long residence time. This provides a platform for a continued and sustained release of the loaded drug to enhance bioavailability, reduce systemic absorption, and the need for frequent administration leading to improved patient compliance [30]. The formulations reported in this study possess some of these properties. However, future efforts will be to increase the gelation temperature of the formulations to broaden their application to meet various extreme ambient temperatures.

**Figure 10:**
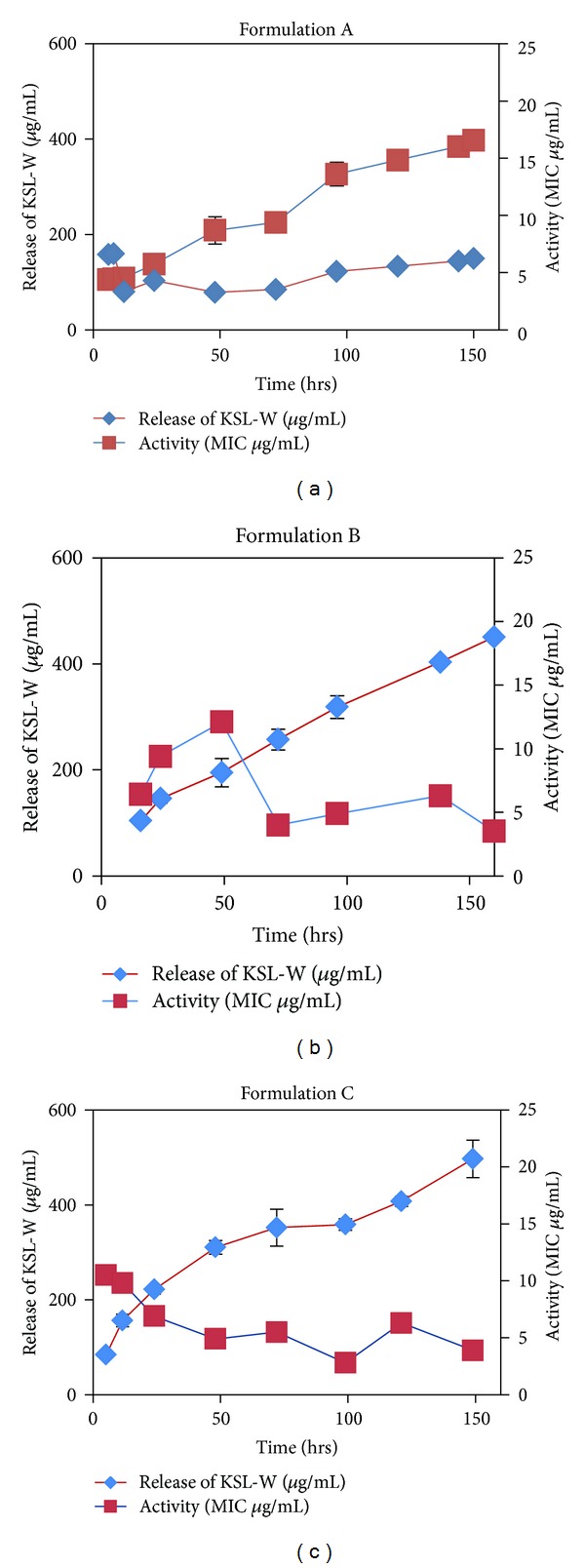
Bactericidal activity of fractions collected from KSL-W PLGA microsphere formulation release using the broth microdilution method. The MIC of KSL-W peptide alone against *S. epidermidis* was 6.25 *μ*g/mL.

Antimicrobial activity of the release fraction collected for each KSL-W microsphere formulation as determined against *staphylococcus epidermidis* using the broth microdilution method is shown in Figure 10. After proper dilution of each collected fraction for the starting concentration in the bactericidal assay, all three microsphere formulations (Formulations A, B, and C) tested showed antimicrobial activity (MIC around 6.25 *μ*g/mL). *S. epidermidis*, one of the most prevalent bacteria found on human skin and mucous membrane microbial flora, has emerged as major source of nosocomial infections of implanted devices. These coagulase-negative staphylococci readily form biofilms on surfaces.

## 4. Summary and Conclusion

Utilizing a thermosensitive gel, the sustained release of an antimicrobial peptide, KSL-W, in porous, hydrophilic, low molecular weight polylactide-co-glycolide microspheres provided antimicrobial activity for up to one week. All three formulations produced showed a similar gelation profile as compared to that of the carrier. Fractions collected from the *in vitro* release media of the three formulations studied showed antimicrobial activity against the targeted microorganism *Staphylococcus epidermidis*. These results suggest that a KSL-W microsphere in 20% Pluronic F127 gel has considerable potential as a delivery system for achieving antimicrobial activity and effective wound healing in combat-related injuries. Formulation B was selected as the choice for further characterization based on the total percent release (Figure 8) which is higher than formulations A & C. In addition, formulation B possesses the smallest particle size among all three formulations (Figure 7).

## Figures and Tables

**Table 1:** KSL-W/PLGA microspheres: composition of formulations and manufacturing parameters.

Formulation	PLGA (mg)	(CH_2_Cl_2_) (mL)	KSL-W (mg)	KSL-W Solvent	PVA % (w/v)	Stirring (°C) Time
A	600	20	200	2 mL DIW* 0.1 mL Tween 20	0.35	25°C 30 m 40°C 1 hr
B	600	20	200	4 mL DIW + 0.1 mL Tween 20	0.35	25°C 30 m 40°C 1 hr
C	600	20	200	3 mL DIW + 0.1 mL Tween 20	0.35	25°C 30 m 40°C 1 hr

*DIW: deionized water.

**Table 2:** KSL-W microsphere properties.

Formulation	Surface morphology	Yield (%)	KSL-W content (%)	Loading efficiency (%)	average size (*µ*m) *n* = 30	% Release at 150 hours	
						In PBS	In Gel
A	Spherical, high surface porosity	75	17	68	46.4 ± 17.9	52.0 ± 2.5	64.4 ± 2.7
B	Spherical, high surface porosity	84	19	77	25.8 ± 14.1	74.8 ± 2.3	76.3 ± 1.2
C	Spherical, high surface porosity	68	20	78	40.0 ± 12.8	53.3 ± 4.5	68.3 ± 3.6

**Table 3:** Gelation temperature (Tg), viscosity, and pH of Formulations.

Formulation code	Gelation temp. (Tg): °C	Viscosity (cp)						pH
		10°C	15°C	20°C	24.6°C	25°C	37°C	
Blank^∗€^	24.6 ± 0.1	39.2 ± 1.5	38.2 ± 0.2	75.0 ± 2.5	103,820.0 ± 16.2	120,216.7 ± 46.4	195,616.7 ± 224.8	6.6 ± 0.2
A^€^	24.7 ± 0.2	40.5 ± 0.2	36.2 ± 0.2	111.0 ± 2.9	117,426.7 ± 90.0	121,533.3 ± 205.5	196,133.3 ± 249.4	6.5 ± 0.1
B^€^	24.6 ± 0.1	41.0 ± 0.5	37.1 ± 0.2	130.0 ± 13.0	110,400.0 ± 294.3	118,485.3 ± 283.8	196,500.0 ± 294.4	6.4 ± 0.2
C^€^	24.7 ± 0.1	34.5 ± 0.6	38.3 ± 0.4	122.7 ± 3.9	122,400.0 ± 374.2	124,216.7 ± 201.4	195,666.7 ± 0.4	6.7 ± 0.1

*Pluronic F127 20% solution.

^*€*^Readings represent the average (±standard deviations) of samples in triplicates.
